# Clinical Significance of UCA1 to Predict Metastasis and Poor Prognosis of Digestive System Malignancies: A Meta-Analysis

**DOI:** 10.1155/2016/3729830

**Published:** 2016-12-15

**Authors:** Xiao-Dong Sun, Chen Huan, Wei Qiu, Da-Wei Sun, Xiao-Ju Shi, Chuan-Lei Wang, Chao Jiang, Guang-Yi Wang, Guo-Yue Lv

**Affiliations:** ^1^Department of Hepatobiliary and Pancreatic Surgery, The First Hospital of Jilin University, Changchun, Jilin Province 130021, China; ^2^Institute of Virology and AIDS Research, The First Hospital of Jilin University, Changchun, Jilin Province 130021, China

## Abstract

*Purpose*. Urothelial carcinoma-associated 1 (UCA1) has been reported to be overexpressed and correlated with progression in various cancers. However, the association between UCA1 expression and some clinicopathological features of digestive system malignancies, such as metastasis and survival, remains inconclusive. Therefore, a meta-analysis was performed to investigate the clinical significance of UCA1 in digestive system malignancies.* Methods*. Relevant literatures were searched in PubMed, Web of Science, Cochrane Library, and Embase databases updated to May 2016.* Results*. A total of 1089 patients from 10 studies were included in this meta-analysis. Meta-analysis results showed that digestive system malignancy patients with UCA1 overexpression were significantly more susceptible to developing lymph node metastasis (LNM) (OR = 1.85, 95% CI: 1.28–2.67) and distant metastasis (DM) (OR = 3.14, 95% CI: 1.77–5.58) and suffer from poor overall survival (OS) (HR = 2.31, 95% CI: 1.89–2.82, univariate analysis; HR = 2.24, 95% CI: 1.69–2.98, multivariate analysis) and poor disease-free survival (DFS) (HR = 2.65, 95% CI: 1.59–4.43, univariate analysis; HR = 2.50, 95% CI: 1.62–3.86, multivariate analysis).* Conclusion*. UCA1 overexpression was correlated with LNM, DM, poor OS, and poor DFS. UCA1 may serve as an indicator for metastasis and poor prognosis in digestive system malignancies.

## 1. Introduction 

Digestive system malignancies have threatened human health seriously. According to the GLOBOCAN estimates, there were about 3.4 million new cases and 2.9 million deaths caused by digestive system malignancies in 2012 worldwide [1]. Although great achievements have been made in therapeutic approaches, such as surgery and chemotherapy, the outcome of digestive system malignancies remains poor. Nowadays, advantage of biomarkers in diagnosis and prognosis of cancers has been suggested, which might provide more precise information for individualized treatment and disease monitoring [2, 3].

Long noncoding RNAs (lncRNAs) are a class of RNAs longer than 200 nt that lack protein-coding capacity [4]. Despite the fact that they used to be regarded as “junk" of genome, increasing number of studies have suggested the contribution of lncRNAs to various biological processes via transcriptional and posttranscriptional regulation [5, 6]. In particular, role of lncRNA in carcinogenesis has been highlighted recently [7]. LncRNAs were found to be dysregulated and function as oncogene or tumor suppressor in various cancers. Furthermore, a growing body of evidence has demonstrated that there was significant association between the expression of lncRNA and the progression of cancer, including clinical-pathological features and survival, indicating that lncRNA can serve as biomarker for cancers. Some lncRNAs, such as HOX transcript antisense RNA (HOTAIR) and metastasis associated lung adenocarcinoma transcript 1 (MALAT1), have been illustrated to potentially predict metastasis and prognosis of digestive system malignancies through meta-analysis [8–10].

Recently, lncRNA urothelial carcinoma-associated 1 (UCA1) has attracted great attention due to its involvement in diverse cancers. UCA1, which was also called cancer upregulated drug resistant (CUDR), was originally identified in bladder transitional cell carcinoma in 2008 and suggested to promote cell proliferation and transformation [11, 12]. So far, the overexpression of UCA1 has been reported in other cancers, especially in digestive system malignancies, including hepatocellular carcinoma [13, 14], gastric cancer [15, 16], colorectal cancer [17–20], pancreatic cancer [21], and esophageal squamous cell carcinoma [22]. Although the association between UCA1 expression and cancer progression was a particular concern for these studies, these results were limited by small sample sizes or inconsistent conclusions. For instance, the patients with high UCA1 level in cancerous tissues were suggested to suffer from elevated lymph node metastasis (LNM) and distant metastasis (DM) rate in numerous cancers [13, 18, 19, 22]; nevertheless, this association has not been detected in other studies [15, 17]. Moreover, the issue of whether the overexpression of UCA1 could predict poor prognosis [16, 20] or not [14] also needs to be clarified. Therefore, to investigate the clinical value of UCA1, we performed this quantitative meta-analysis to assess the correlation of UCA1 expression with metastasis and prognosis in digestive system malignancies.

## 2. Material and Methods

### 2.1. Literature Search Strategy

All literatures investigating the association of lncRNA UCA1 with metastasis and prognosis of digestive system malignancies were searched in PubMed, Web of Science, Cochrane Library, and Embase databases updated to May 2016. Search terms are as follows: “UCA1" or “urothelial carcinoma-associated 1" or “CUDR" or “cancer up-regulated drug resistant", “cancer" or “carcinoma" or “tumor" or “neoplasm", and “survival” or “prognosis" or “prognostic" or “progression" or “recurrence" or “outcome" or “metastasis" or “clinicopathological". The searching strategy used in PubMed was “((((((UCA1 [Title/Abstract]) OR urothelial carcinoma-associated 1 [Title/Abstract]) OR CUDR [Title/Abstract]) OR cancer up-regulated drug resistant [Title/Abstract])) AND ((((cancer) OR carcinoma) OR tumor) OR neoplasma)) AND ((((((((survival) OR prognosis) OR prognositic) OR progression) OR recurrence) OR outcome) OR metastasis) OR clinicopathological)". In addition, the references in retrieved articles were screened manually for potential relevant studies.

### 2.2. Selection and Exclusion Criteria

The articles collected were considered eligible if they met the inclusion criteria: (1) articles were investigating the association of UCA1 with progression of digestive system malignancies; (2) the expression levels of UCA1 in primary cancerous tissues were measured; (3) patients were grouped according to the expression levels of UCA1; (4) related clinicopathological parameters were described. Exclusion criteria are the following: (1) duplicate publications; (2) reviews, letters, comments, and conference articles; (3) studies focusing on UCA1 in other types of cancers, rather than digestive system malignancies; (4) studies using cells lines or animals, rather than cancer patients; (5) studies without usable data. Regarding multiple publications from the same medical center, only the most recent or the most complete study was included in the meta-analysis.

### 2.3. Data Extract

Two investigators (Xiao-Dong Sun, Chen Huan) extracted data from the eligible studies independently, according to the inclusion and exclusion criteria. For disagreements, a consensus was achieved by a third investigator (Wei Qiu). The following information was collected from each eligible study: first author, publication year, region of patients, cancer type, total number of patients, detecting method of UCA1 expression, cut-off value of grouping, number of patients in high/low UCA1 expression group, number of patients with LNM and DM in each group, follow-up time, study endpoint, survival analysis method (multivariate or univariate), and hazard ratio (HR) with 95% confidence interval (CI) for overall survival (OS) or disease-free survival (DFS). When the HRs and their 95% CIs were given in the articles, these data were extracted directly. If the prognosis was plotted as Kaplan–Meier curve, data was digitized by the software Engauge Digitizer version 4.1 (http://digitizer.sourceforge.net/) and calculated as described [23].

### 2.4. Quality Assessment

Quality assessment of the included studies was performed according to the Newcastle-Ottawa Scale (NOS) criteria [24]. The NOS criteria is scored based on three aspects (subject selection, comparability of subject, and clinical outcome) with the final scores ranging from 0 to 9, and a score ≥6 indicates a high quality.

### 2.5. Data Analysis

To assess the heterogeneity among the included studies, *χ* ^2^-based *Q* test and *I* ^2^ statistics were used. When heterogeneity was significant (*I* ^2^ > 50% or *P* < 0.10 for *χ* ^2^), a random effects model was used; otherwise, fixed effects model was adopted. The potential publication bias was evaluated using a “funnel plot" as well as Begg's and Egger's test.

The meta-analysis was performed through using Stata SE12.0 (Stata Corporation). All *P* values were two-sided, and *P* < 0.05 was considered statistically significant.

## 3. Results

### 3.1. Literature Information

**Figure 1:**
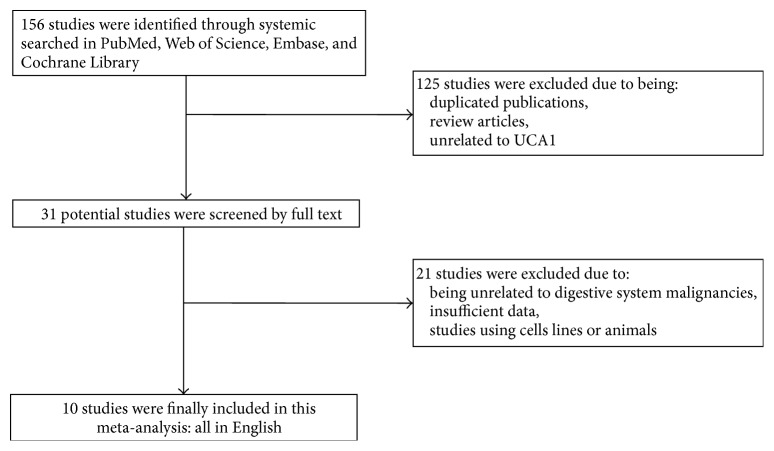
Flow diagram of searching relevant studies used in this meta-analysis.

A total of 156 records were retrieved by searching the databases of PubMed, Web of Science, Cochrane Library, and Embase. After screening the title and abstract, 125 articles were excluded because they were either duplication or reviews or about other lncRNAs. For the thirty-one articles remaining, full text was assessed carefully and 21 were excluded for their insufficient data, or for they focused on the level of UCA1 from other cancers rather than digestive system malignancies. Finally, 10 articles comprising 1089 patients were identified as eligible and included in the present meta-analysis. The flow diagram was shown in Figure 1.

### 3.2. Study Characteristics

The baseline characteristics of the included studies were summarized in Table 1. The articles were published between 2014 and 2016 with sample sizes ranging from 54 to 240. Nearly all of them were conducted in Asia, 8 studies in China, 1 study in Korea, and 1 in USA. To detect the UCA1 expression, quantitative reverse transcription-polymerase chain reaction (RT-PCR) was used in 8 studies, Illumina expression beadchip was used in 1 study, and Affymetrix 2.0 microarray was used in 1 study. The cut-off value for UCA1 expression was unavailable in 2 studies, and the remaining 8 were based on median value of UCA1 level (in 5 studies), mean value of UCA1 level (in 2 studies), and fourth quartile of UCA1 level (in 1 study). According to the NOS criteria, all of the included studies got 7 scores or more, indicating their high methodological quality (Table 2).

### 3.3. Meta-Analysis for OS

#### 3.3.1. Association between UCA1 and Metastasis

**Figure 2:**
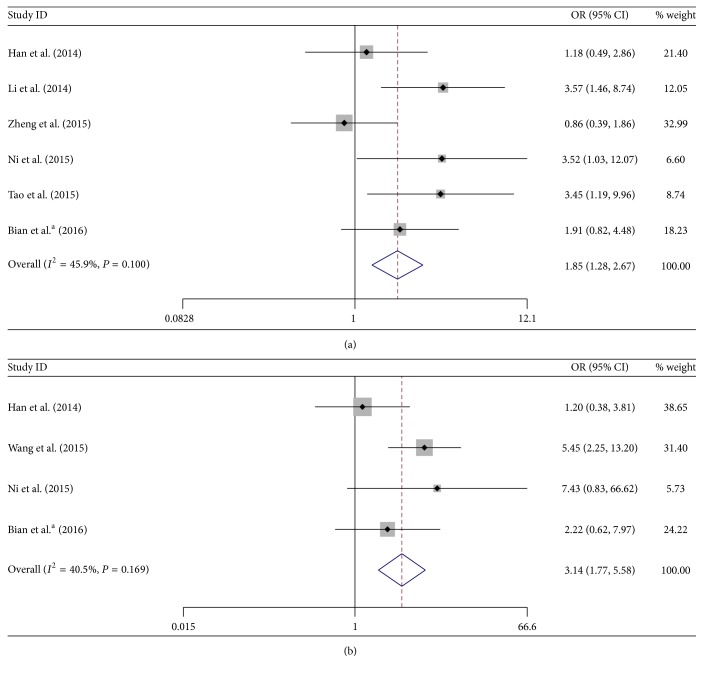
Forest plots of odds ratios (ORs) for the association between UCA1 expression and lymph node metastasis (LNM) (a) and distant metastasis (DM) (b).

In total 6 studies including 506 cases reported the association of UCA1 with LNM of digestive system malignancies. Since there was no significant heterogeneity among these studies (*I* ^2^ = 45.9% and *P* = 0.100), the fixed model was adopted. The pooled OR with 95% CI indicated that digestive system malignancy patients with high UCA1 level in tumor tissues were more susceptible to developing LNM (OR = 1.85, 95% CI: 1.28–2.67, *P* = 0.001) (Figure 2(a)).

Moreover, there were four studies comprising 322 patients that investigated correlation of UCA1 expression and the occurrence of DM in digestive system malignancies. There was also no significant heterogeneity among these studies (*I* ^2^ = 40.5% and *P* = 0.169), so the fixed model was applied to calculate the pooled OR and its 95% CI. The result showed that increased UCA1 expression was significantly correlated with DM (OR = 3.14, 95% CI: 1.77–5.58, *P* = 0.000) (Figure 2(b)).

Taken together, the results above showed that UCA1 overexpression was significantly correlated with LNM and DM in digestive system cancer patients, suggesting that UCA1 may serve as an indicator for metastasis of digestive system malignancies.

#### 3.3.2. Association between UCA1 and OS

**Figure 3:**
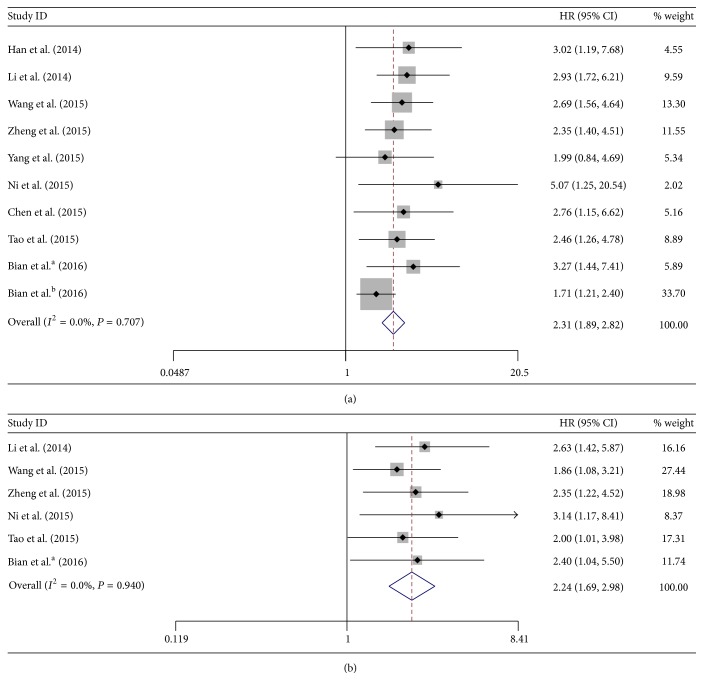
Forest plots of hazard ratios (HRs) for the association between UCA1 expression with overall survival (OS) from univariate analysis results (a) and OS from multivariate analysis results (b).

On one hand, 9 studies with a total number of 1012 patients investigated the association between UCA1 expression and OS through univariate analysis. The fixed model was used to assess the pooled HR and its 95% CI since no significant heterogeneity was found among these studies (*I* ^2^ = 0%, *P* = 0.707). We found that high UCA1 level was significantly associated with poor OS (HR = 2.31, 95% CI: 1.89–2.82, *P* = 0.000) (Figure 3(a)). On the other hand, 6 studies with a total number of 524 patients investigated the association between UCA1 expression and OS through multivariate analysis. Since there was no significant heterogeneity among these studies (*I* ^2^ = 0%, *P* = 0.940), the fixed model was used to assess the pooled HR and its 95% CI. We found that UCA1 overexpression was also significantly associated with poor OS (HR = 2.24, 95% CI: 1.69–2.98, *P* = 0.000) (Figure 3(b)).

Results from the above analysis indicated that high expression of UCA1 was significantly correlated with poor OS in digestive system cancer patients, suggesting that UCA1 was an indicator of decreased survival rate in digestive system malignancies.

#### 3.3.3. Association between UCA1 and DFS

**Figure 4:**
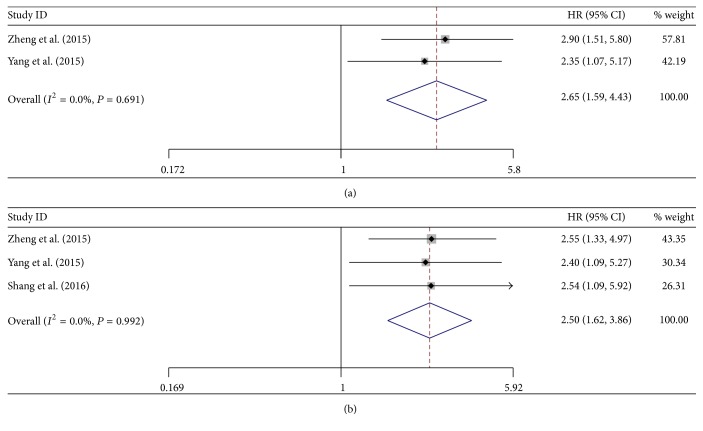
Forest plots of hazard ratios (HRs) for the association between UCA1 expression with disease-free survival (DFS) from univariate analysis results (a) and DFS from multivariate analysis results (b).

Totally, there were 3 studies including 429 patients investigating the prognostic value of UCA1 on DFS in the form of either univariate or multivariate analysis. Since no significant heterogeneity was found among these studies (*I* ^2^ = 0.0%, *P* = 0.691, univariate analysis; *I* ^2^ = 0.0%, *P* = 0.992, multivariate analysis), fixed-effect model was adopted to calculate the pooled HRs and their 95% CIs. The results showed that increased UCA1 expression was also significantly associated with poor DFS (HR = 2.65, 95% CI: 1.59–4.43, *P* = 0.000, univariate analysis; HR = 2.50, 95% CI: 1.62–3.86, *P* = 0.000, multivariate analysis) (Figures 4(a) and 4(b)), indicating that increased UCA1 expression was an indicator of early tumor recurrence in digestive system cancer patients.

### 3.4. Publication Bias

**Figure 5:**
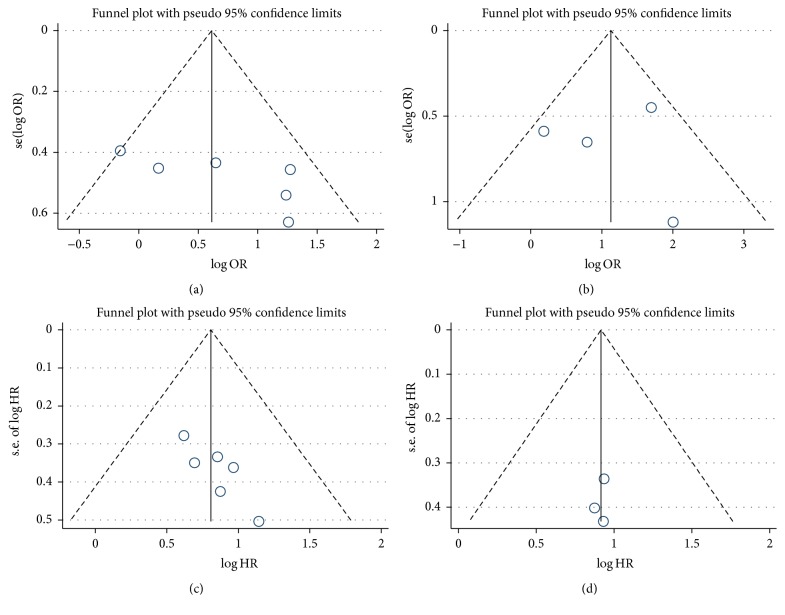
Funnel plots for the meta-analysis with lymph node metastasis (LNM) (a), distant metastasis (DM) (b), overall survival (OS) from multivariate analysis results (c), and disease-free survival (DFS) from multivariate analysis results (d).

In this meta-analysis, both Begg's and Egger's *P* value tests were used to assess the potential publication bias. No publication bias was found in the studies with LNM (*P* = 0.188, 0.109), DM (*P* = 1.000, 0.949), or OS (*P* = 0.128 for Begg's test, univariate analysis) or DFS (*P* = 0.602, 0.746, multivariate analysis). However, publication bias was found in the studies with OS (*P* = 0.003 for Egger's test, univariate analysis; *P* = 0.039, 0.035, multivariate analysis). Besides, the funnel plots for LNM (Figure 5(a)), DM (Figure 5(b)), OS from multivariate analysis (Figure 5(c)), and DFS from multivariate analysis (Figure 5(d)) were largely symmetrical. Therefore, we speculate that most of our meta-analysis results are reliable.

## 4. Discussion

Digestive system malignancies constitute a major part of human cancers [1]. Their rapid progression and poor outcome make it necessary and essential to identify biomarkers, which could improve the diagnosis and therapy by providing more precise and valuable information. UCA1, a lncRNA located at 19p13.12, has been found to be upregulated and exert oncogenic function in digestive system malignancies. In colorectal carcinoma, for example, overexpression of UCA1 was illustrated to promote proliferation and cell cycle progression and inhibit apoptosis, whereas suppression of UCA1 inhibited cell proliferation and cell cycle progression and facilitated apoptosis [17]. Regarding the mechanism, UCA1 can act as a competing endogenous RNA (ceRNA) by directly binding to microRNAs (miRNAs). In hepatocellular carcinoma, upregulated UCA1 contributes to the progression of cancer by counteracting the inhibitory effect of miR-216b and activating the FGFR1/ERK signaling pathway [13]. In colorectal cancer, UCA1 was found to function as an endogenous sponge by directly binding to miR-204-5p and promote the expression of a new target of miR-204-5p, CREB1 [20]. The results above suggested that UCA1 plays an important role during the carcinogenesis and may serve as a potential target for treatment of digestive system malignancies. Recently, several studies have investigated the clinicopathological value of UCA1 expression in digestive system malignancies. However, the sample sizes in most studies are small. Besides, it is inconclusive about the association between UCA1 expression and progression of digestive system malignancies, such as metastasis and survival. Therefore, we conducted this meta-analysis with the aim of clarifying the clinical significance of UCA1 expression in digestive system malignancies.

To the best of our knowledge, this is the first meta-analysis to investigate the association of UCA1 expression with the metastasis and prognosis of digestive system malignancy patients. We included 10 studies with a total of 1089 patients. The pooled ORs with their 95% CIs showed that high UCA1 expression was significantly associated with LNM and DM, indicating that UCA1 was an indicator for metastasis of digestive system malignancies. Moreover, the pooled HRs with their 95% CIs showed that UCA1 overexpression was also significantly correlated with both poor OS and poor DFS, indicating that UCA1 overexpression may serve as an indicator of poor survival rate and high recurrence rate of digestive system malignancies, respectively. What is more, as the lncRNA can be secreted by cancer cells or released into the circulation from dead cancer cells, it has been reported that UCA1 level in plasma significantly decreased 14 days after surgery of colon cancer [19]. To some extent, it demonstrated the correlation between UCA1 overexpression and the aggressive behavior of digestive system malignancies, which was concordant with our conclusion. Taken together, UCA1 could serve as a promising biomarker for monitoring the progression of digestive system malignancies.

Nevertheless, there are several limitations in this meta-analysis. Firstly, most of the included studies were performed in the population from Asian countries rather than worldwide population; our results should be substantiated by additional studies in other races. Secondly, publication bias was observed in the studies with OS, which may be due to the fact that some studies were not included in this meta-analysis for their insufficient data or that some studies reported the correlation in one analytic method. Thirdly, the included studies were of small sample size as well as different cut-off value of UCA1 expression, which may generate errors by variation. Based on these limitations, the pooled ORs and HRs with their 95% CIs calculated in this meta-analysis may be just estimations.

## 5. Conclusion

In conclusion, our meta-analysis showed that UCA1 overexpression was correlated with LNM, DM, poor OS, and poor DFS in digestive system malignancy patients. Therefore, UCA1 may serve as an indicator of metastasis and prognosis in digestive system malignancies.

## Figures and Tables

**Table 1:** Characteristics of included studies in this meta-analysis.

First author [ref.]	Year	Cancer	Country	Sample size	Methods for UCA1 detecting	Cut-off value for UCA1	UCA1 expression						Study endpoints	(HR, 95% CI)	Data source	Follow-up time (months)
							High expression	High with LNM	High with DM	Low expression	Low with LNM	Low with DM				
Han [17]	2014	CRC	China	80	qRT-PCR	Mean value	37	17	7	43	18	7	OS	OS (U), 3.02 (1.19–7.68)	Curve	Mean 42.6

Li [22]	2014	ESCC	China	90	qRT-PCR	Mean value	41	22	NA	49	12	NA.	OS	OS (U), 2.93 (1.72–6.21)	Direct	Median 43
														OS (M), 2.63 (1.42–5.87)	Direct	

Wang [13]	2015	HCC	China	98	qRT-PCR	Median value	49	NA	30	49	NA	11	OS	OS (U), 2.69 (1.56–4.64)	Direct	Up to 60
														OS (M), 1.86 (1.08–3.21)	Direct	

Zheng [15]	2015	GC	China	112	qRT-PCR	Median value	56	35	NA	56	37	NA	OS, DFS	OS (U), 2.35 (1.40–4.51)	Direct	Up to 60
														OS (M), 2.35 (1.22–4.52)	Direct	
														DFS (U), 2.90 (1.51–5.80)	Direct	
														DFS (M), 2.55 (1.33–4.97)	Direct	

Yang [14]	2015	HCC	Korea	240	Illumina expression beadchip	Median value	120	NA	NA	120	NA	NA	OS, DFS	OS (U), 1.99 (0.84–4.69)	Direct	Up to 120
														DFS (U), 2.35 (1.07–5.17)	Direct	
														DFS (M), 2.40 (1.09–5.27)	Direct	

Ni [18]	2015	CRC	China	54	qRT-PCR	Median value	27	12	6	27	5	1	OS	OS (U), 5.07 (1.25–20.54)	Available data	Up to 50
														OS (M), 3.14 (1.17–8.41)	Available data	

Chen [21]	2015	PDAC	USA	63	Affymetrix 2.0 microarray	NA	NA	NA	NA	NA	NA	NA	OS	OS (U), 2.76 (1.15–6.61)	Available data	Median 21
Tao [19]	2015	CRC	China	80	qRT-PCR	Fourth quartile	20	13	NA	60	21	NA	OS	OS (U), 2.46 (1.26–4.78)	Direct	Up to 72
														OS (M), 2.00 (1.01–2.98)	Direct	

Bian [20]^*∗*^	2016	CRC	China	90^a^	qRT-PCR	Median value	45	30	8	45	23	4	OS	OS (U), 3.27 (1.44–7.41)	Direct	Up to 80
														OS (M), 2.40 (1.04–5.50)	Direct	
				105^b^			NA	NA	NA	NA	NA	NA	OS	OS (U), 1.71 (1.21–2.40)	Curve	Up to 120

Shang [16]	2016	GC	China	77	qRT-PCR	NA	NA	NA	NA	NA	NA	NA	DFS	DFS (M), 2.54 (1,09–5.92)	Direct	Up to 72

NA, not available; qRT-PCR, quantitative reverse transcription-polymerase chain reaction; LNM, lymph node metastasis; DM, distant metastasis; OS, overall survival; DFS, disease-free survival; U, univariate analysis; M, multivariate analysis; Curve, Kaplan–Meier curve; ^*∗*^this study included two cohorts of CRC patients; we named them Bian et al.^a^ and Bian et al.^b^, respectively, in the following analysis.

**Table 2:** Newcastle-Ottawa quality for included studies in this meta-analysis.

First author and year [ref.]	Selection (score)				Comparability (score)		Outcome (score)			Total score
	Representativeness of exposed	Selection of nonexposed	Ascertainment of exposure	No interest before study	Study design (cohort study)	Control for other confounding factors	Assessment of outcome	Follow-up time long enough (>5 years )	Adequacy number of follow-ups (>80% )	
Han 2014 [17]	1	1	1	0	1	0	1	1	1	7
Li 2014 [22]	1	1	1	0	1	1	1	1	1	8
Wang 2015 [13]	1	1	1	1	1	1	1	1	1	9
Zheng 2015 [15]	1	1	1	0	1	1	1	1	1	8
Yang 2015 [14]	1	1	1	0	1	1	1	1	1	8
Ni 2015 [18]	1	1	1	0	1	1	1	0	1	7
Chen 2015 [21]	1	1	1	0	1	1	1	1	1	8
Tao 2015 [19]	1	1	1	0	1	1	1	1	1	8
Bian 2016 [20]	1	1	1	0	1	1	1	1	1	8
Shang 2016 [16]	1	1	1	0	1	1	1	1	1	8
